# Treatment of Aphthæ by Sulphuric Acid

**Published:** 1846-10

**Authors:** 


21. *Treatment of Aphthæ by Sulphuric Acid.*—Prof. Lippich, of Padua, has recommended the following liniment in the treatment of aphthæ:—Honey, 15 parts; diluted sulphuric acid, 1 part, by weight. The ulcerated surfaces should be occasionally brushed over with this liniment by means of a camel's-hair pencil. The proportion of sulphuric acid may be increased if the case is obstinate.—*Med. News.*

22. *Singultus.*—M. Rostan has recently employed with success strong pressure on the epigastrium in several cases of severe hiccup.—*Ibid.*

